# Multi-objective optimization of table tennis serve kinematic parameters using an improved particle swarm optimization algorithm

**DOI:** 10.3389/fphys.2026.1932912

**Published:** 2026-09-04

**Authors:** LinKun Wang, HuiJun Li

**Affiliations:** School of Physical Education, Inner Mongolia University, Hohhot, China

**Keywords:** multi-objective optimization, pareto optimality, particle swarm optimization, precision training, serve kinematics, table tennis

## Abstract

**Background:**

Table tennis serve is the only technical action fully controlled by athletes during competition. Its biomechanical characteristics directly determine the coherence of the first three strokes, the initiation of active offense, and overall match performance. Conventional serve training relies predominantly on coaches**’** empirical judgment and athletes**’** subjective perception, which cannot satisfy the growing demand for refined optimization of multi-dimensional biomechanical parameters including spin, speed and placement.

**Objective:**

Existing studies remain limited in elucidating the coupling relationships among serve biomechanical parameters, establishing a comprehensive evaluation framework that balances competitive efficacy and motor executability, and deploying intelligent methods for individualized serve parameter optimization. This study aims to construct a data-driven multi-objective optimization framework for table tennis serve kinematic and performance parameters.

**Methods:**

Five core kinematic and serve-control variables were selected as decision parameters: racket face angle, ball contact height, hitting timing, swing velocity, and landing coordinates. A multi-objective evaluation model was developed, integrating spin intensity, ball speed, placement stability, return suppression effect and motor adjustment cost. An improved particle swarm optimization (PSO) algorithm embedded with dynamic inertia weight, constraint correction, non-dominated sorting and crowding distance mechanisms was applied to generate Pareto optimal solution sets and screen athlete-specific serve schemes. The calibration dataset included 42 athletes (1,512 trials), and the validation dataset included 18 athletes (648 trials). A separate serve–return block involved 18 independent receivers, and individualized recommendations were evaluated in a 4-week pre–post intervention.

**Results:**

The improved PSO-Pareto method outperformed conventional algorithms in convergence efficiency, solution set diversity and distribution uniformity. Six categories of Pareto-optimal serve schemes with distinct biomechanical and tactical orientations were identified. Individualized parameter recommendations showed descriptive improvements in overall serve quality, landing-point accuracy, return-suppression effect and third-stroke connection performance across different athlete types.

**Conclusions:**

The proposed framework enables quantitative and individualized optimization of table tennis serve kinematic parameters based on measured movement and performance characteristics. It provides a methodological basis for data-driven and precision-oriented serve training, and offers a reference for biomechanical parameter optimization of other sport techniques.

## 1 Introduction

Table tennis serve is the only technical action fully controlled by athletes during competition, whose quality directly determines the coherence of the first three strokes, the initiation of active offense, and the opponent’s return decision-making. As modern table tennis imposes increasingly high demands on speed, spin, placement and rhythm, serve training has transitioned from empirical repetition to refined analysis underpinned by sports biomechanics and technical parameterization (Bańkosz and Winiarski, 2020; Bańkosz et al., 2020; Wong et al., 2020). Extant kinematic studies have demonstrated that table tennis stroke movements rely on coordinated activation of the upper limbs, trunk and lower limbs, and that technical gaps between athletes of different levels are manifested in stroke trajectory, racket-face control and motion stability (Cao et al., 2020; Djokic et al., 2020). Beyond biomechanical characterization, research has underscored the tactical role of serve as the launch point for subsequent tactical organization rather than an isolated action (Xia et al., 2020). Kinematic variations across grip patterns and spin categories further indicate that serve design should be tailored to athletes’ individual technical styles (Ferrandez et al., 2021). Meanwhile, empirical evidence has validated that multi-ball training and specialized perceptual training enhance hitting performance in young athletes, highlighting the critical value of targeted parameter design for technical improvement (He et al., 2021).

Regarding performance evaluation, the field has moved beyond single metrics such as speed or spin, recognizing that serve quality should be comprehensively gauged by placement precision, return restriction, and subsequent offensive gains (Fuchs and Lames, 2021; Huang et al., 2021; Malagoli Lanzoni et al., 2021). Multi-index evaluation models have been developed to quantify the contribution of technical actions to match performance, laying a foundation for objective assessment of serve value (Yu and Gao, 2022; Zhou and Zhang, 2022). The rise of data-driven methods including neural network-based technical and tactical diagnosis and a three-stage performance evaluation framework further makes it possible to identify key determinants of match outcomes, providing new methodological support for service quality analysis (Agarwal et al., 2022; Ariyaratne and Silva, 2022). Nevertheless, serve optimization is inherently a multi-objective problem. Athletes seek to simultaneously boost ball speed, amplify spin, tighten placement control, enhance motion concealment, and preserve action stability, but these objectives often involve inherent trade-offs (Pradas de la fuente et al., 2023). Excessive pursuit of ball speed may compromise placement accuracy; over-strong spin may increase the risk of action exposure; and overemphasis on concealment may reduce serve stability (Grycan et al., 2023). Traditional single-objective screening easily leads to misaligned training goals (Song et al., 2023; Yin et al., 2023). Pareto optimality theory resolves this dilemma by generating a non-dominated solution set to delineate trade-offs among competing objectives, enabling parameter selection adapted to match scenarios, personal technical styles and opponent characteristics (Shu et al., 2023).

Particle swarm optimization (PSO), characterized by a simple structure, high search efficiency, and suitability for continuous-variable optimization, has shown potential in sports parameter optimization. Metaheuristic optimization has also been introduced into biomechanics to select informative gait features from multidimensional kinematic and kinetic data, demonstrating its value in identifying biomechanically meaningful movement patterns (Xu et al., 2024). Improved PSO variants further provide an effective means of balancing convergence and solution diversity in complex multi-objective problems (Agarwal et al., 2022; Pradas de la fuente et al., 2023; Shi et al., 2024). However, their application to the multi-objective optimization of table tennis serve parameter combinations remains limited. While intelligent algorithms have been widely adopted in table tennis for action recognition, match analysis and technical classification (Shi et al., 2024; Song et al., 2024; Yenduri et al., 2024), their application to multi-objective optimization of serve parameter combinations remains largely unexplored. Despite these advances, existing studies suffer from three key limitations: insufficient investigation of the coupling relationships among serve speed, spin, placement, hitting posture and stability, the absence of a comprehensive evaluation model integrating threat level, motion stability, action concealment and individual adaptability, and the underuse of intelligent algorithms for multi-objective serve parameter optimization, with conventional methods unable to capture trade-offs across multiple training objectives.

This study constructs a multi-objective optimization model for table tennis serve parameters, and introduces an improved particle swarm optimization algorithm to generate the Pareto optimal solution set. This work aims to identify serve parameter combinations that balance competitive efficacy and individual fitness, providing quantitative support for the refined, individualized and digital development of serve training strategies.

## 2 Methods

### 2.1 Participants

A total of 60 competitive table tennis athletes (36 males and 24 females) voluntarily participated in this study. The participants had a mean age of 21.4 ± 2.8 years, a mean height of 172.5 ± 8.3 cm, a mean body mass of 65.2 ± 9.6 kg, and an average systematic training experience of 8.6 ± 3.2 years. According to the official athlete classification system, the sample included 8 national-level athletes, 22 first-class athletes, and 30 second-class athletes. Among them, 52 athletes were right-handed and 8 were left-handed.

An additional receiver cohort of 18 competitive athletes (10 males and 8 females; mean age, 21.8 ± 2.5 years) participated exclusively in the serve–return validation block. The receiver cohort included 4 national-level, 8 first-class, and 6 second-class athletes. No participant served in both the server and receiver roles.

The inclusion criteria were as follows: (1) at least 5 years of systematic table tennis training; (2) regular participation in technical training or competitive matches; (3) the ability to consistently perform short backspin, long backspin, sidespin, and placement-directed serves; and (4) no musculoskeletal injury affecting serve execution during the previous 6 months. Participants were excluded when they experienced pain or physical discomfort during testing, failed to complete the prescribed serve protocol, or produced incomplete motion and ball-trajectory recordings.

Before data collection, all participants were informed of the experimental procedures, potential risks, and data-processing methods. Written informed consent was obtained from each participant. The experimental protocol was approved by the Ethics Committee of the School of Physical Education, Inner Mongolia University and was conducted in accordance with the relevant ethical guidelines for research involving human participants.

### 2.2 Experimental apparatus and testing conditions

The experiment was conducted in an indoor table tennis laboratory under standardized environmental conditions. The laboratory temperature was maintained at 22 ± 1 °C, the relative humidity was 50 ± 5%, and no perceptible airflow was present during data collection. A standard competition table (DHS T1223, Double Happiness Sports, China) and standardized 40+ table tennis balls (DHS 40+, Double Happiness Sports, China) were used throughout the experiment. The lighting conditions and equipment arrangement remained unchanged for all participants.

Racket and ball trajectories were recorded using 4 high-speed cameras (Phantom VEO 710, Vision Research, USA) operating at a sampling frequency of 500 frames/s, a spatial resolution of 1280 × 800 pixels, and a shutter speed of 1/2000 s. The cameras were positioned at 2.5 m from the table and at a height of 1.5 m, with viewing angles selected to capture the sagittal and frontal planes of the serving action. Swing velocity was measured using a wearable inertial measurement unit system (Xsens MTw Awinda, Xsens Technologies, Netherlands) at a sampling frequency of 100 Hz. Ball velocity was measured using a radar speed gun (Stalker Sport 2, Applied Concepts, USA), with a reported measurement accuracy of ±0.1 m/s. Ball spin was obtained using a high-speed camera-based marker-tracking method at 500 Hz, while landing coordinates were identified using a camera-based automatic tracking system (Qualisys Track Manager, Qualisys, Sweden) with a spatial accuracy of 0.5 cm.

All acquisition devices were synchronized using a hardware trigger synchronization unit (Qualisys Sync Unit, Qualisys, Sweden). Before each testing session, the measurement space was calibrated using a three-dimensional calibration frame (Peak Performance Technologies, USA). The origin of the coordinate system was defined at the midpoint of the table’s end line on the server’s side, with the x-axis representing the lateral direction and the y-axis representing the longitudinal direction. The mean spatial calibration error was 0.3 cm. Equipment calibration was repeated whenever the camera position or experimental arrangement was changed. All participants used the same standardized racket and rubber configuration during testing to minimize equipment-related variability.

### 2.3 Experimental protocol and data preprocessing

Participants were instructed to avoid high-intensity training for 24 h before testing. Each testing session began with a standardized 15-min warm-up consisting of general upper-limb movements, footwork exercises, and serve-specific practice. Participants subsequently completed 5 familiarization trials to adapt to the laboratory environment and measurement equipment.

The formal testing protocol included short backspin, long backspin, sidespin, and placement-directed serves. Each participant completed 10 valid trials under each serve condition, resulting in 40 planned trials per participant. The order of the serve conditions was randomized using a computer-generated block randomization sequence to reduce potential order and fatigue effects. A rest interval of 30 s was provided between consecutive trials, and a rest period of 3 min was provided between serve blocks.

A trial was considered valid when the serve complied with the official table tennis service rules, the ball landed within the prescribed target area, and complete synchronized data were obtained from all recording devices. Trials were excluded when the ball failed to land on the table, the participant lost balance or interrupted the movement, equipment synchronization failed, or the recording contained missing frames. A total of 2400 serve trials were collected. After quality control, 2160 trials were retained, corresponding to a valid-trial rate of 90.0%.

Raw trajectory signals were processed using MATLAB R2023b (MathWorks, USA). Missing or incomplete trials were removed before analysis. Continuous trajectory data were filtered using a 4th-order Butterworth low-pass filter with a cutoff frequency of 12 Hz. Outliers were identified using the interquartile-range criterion (values exceeding 1.5 × IQR beyond Q1 or Q3) and were verified against the original video recordings before exclusion. Variables with different physical units were transformed using Z-score normalization before being entered into the multi-objective optimization model.

To prevent participant-level information leakage, trials from the same athlete were assigned to the same data partition. The calibration dataset was used to estimate the biomechanical fitting coefficients and determine the feasible parameter ranges, whereas the validation dataset was used to evaluate the optimization results and individualized recommendation effects. The calibration partition comprised 42 athletes and 1,512 retained trials, whereas the validation partition comprised 18 athletes and 648 retained trials.

#### 2.3.1 Serve–return validation protocol and receiver-side outcome coding

A separate serve–return validation block was conducted with the 18 independent receivers described above. Each of the 60 servers faced 3 randomly assigned receivers, producing 180 server–receiver pairings. The block used the same four serve categories as the main protocol, with 6 trials per serve category for each pairing. Of 4,320 planned trials, 3,980 valid trials were retained, corresponding to a valid-trial rate of 92.1%.

Server–receiver pairings and serve orders were block-randomized. Receivers were blinded to spin using an opaque screen. Rest intervals were 30 s between trials and 3 min between blocks. Receiver-side variables included the initial three-dimensional ready posture, forehand or backhand receiving side, distance from the table (0.5–1.0 m), lateral movement range (0–1.2 m), and tactical anticipation category (0, no cue; 1, partial cue; 2, full cue).

ErrRate was calculated as the number of direct return errors divided by valid serves after excluding lets; the denominator was 3,980 serves. PassiveRate was calculated among successful returns. A return was classified as passive when attacking intent was below 30%, the trajectory showed a high arc, or a forced lob occurred. Third-stroke connection was coded as successful when the server initiated an attack within two shots after the return, as failed when a forced error or passive block occurred, and as no opportunity when the return had already ended the rally. Two ITTF-certified coaches coded synchronized video independently, and disagreements were resolved by consensus. Cohen’s κ was 0.88 for ErrRate and 0.82 for PassiveRate. Athlete-level separation was maintained between calibration and validation data. Complete operational definitions are provided in **Supplementary Appendix Table A4**.

#### 2.3.2 Individualized recommendation intervention and pre–post evaluation

At intervention entry, the 60 serving athletes were assigned using a prespecified technical-profile rule to the following groups: speed-dominant (n = 12), spin-dominant (n = 12), landing-point control (n = 10), attack-connection (n = 10), motion-robustness (n = 8), and balanced-overall (n = 8). The pretest was conducted 1 week before the intervention and comprised 40 trials per athlete under the same equipment, environment, target areas, and receiver-matching conditions used at posttest. Pareto-optimal parameter ranges were selected through algorithm–coach consensus, displayed through visual dashboards, and reviewed by two coaches.

Training lasted 4 weeks, with 3 sessions per week and 45 min per session. Each session included 80 recommendation-specific serves arranged in 4 blocks of 20, with 30 s between blocks. Real-time kinematic feedback was provided after each block. The adherence threshold was at least 80%, and mean attendance was 95%.

Posttest assessment occurred 3 days after the final session and used the same 18 receivers, matched by competitive level, with standardized receiving posture and randomized order. Assessors were blinded to the test occasion. Primary outcomes were serve quality, landing-point hit rate, return-suppression effect, and third-stroke connection success, each based on 40 valid trials per athlete. Two athletes withdrew because of mild shoulder strains, leaving 58 completers; no serious adverse events occurred. Missing trial-level data were below 2%, and the manuscript reports descriptive pre–post changes without inferential testing. Complete intervention details are provided in **Supplementary Appendix Table A6**.

### 2.4 Construction of serve parameter system and variable constraint setting

The construction of table tennis serving parameter system is the basis of multi-objective optimization model. The service is determined by the racket angle, the height of the ball, the hitting time, the swing speed and the control of the landing point. Due to the differences in technical characteristics and tactical needs of different athletes, it can not be measured only by speed or rotation. Relevant studies have shown that athletes of different genders and technical types have differences in serving and the connection strategy of the first three boards, so the parameter modeling needs to take into account the action execution conditions and tactical application requirements (Ma and Tong, 2024). This study constructs a computable serve parameter vector to provide variable basis for algorithm optimization. Since particle swarm optimization algorithm may have premature convergence or unenforceable solution in complex parameter space, it is necessary to combine dynamic weight, boundary constraint and other mechanisms to improve the optimization effect (Malagoli Lanzoni et al., 2024). The multi-objective optimization research further points out that the algorithm needs to take into account the search efficiency, diversity of solution sets and constraint feasibility (Han et al., 2024). Therefore, by establishing the parameter system and setting the feasible region limit, this study screened the optimal combination in line with the sports law and training practice. The mathematical formulation and optimization procedure are presented in Equations 1–7.

(1) $$X={\left[\theta ,h,t,v_{s},x,y\right]}^{T}$$

As shown in Formula 1, where: *θ*: the angle between the beat plane and the vertical direction reflects the tilt degree of the beat plane; *h*: the height of the ball when touching the ball, which affects the flight trajectory; *t*: the hitting time determines the motion state of the ball when touching the ball; *v_s_*: swing speed, associated with ball speed and rotation; *x*: the horizontal position of the contact point affects the left and right distribution of the impact point; *y*: the vertical position of the contact point affects the front and rear distribution of the impact point.

(2) $$\Omega =\left\{X/\theta \in \left[15{}^{\circ},60{}^{\circ}\right],h\in \left[0.15,0.35\right],t\in \left[-0.1,0.1\right],v_{s}\in \left[v_{s,g}^{\min},v_{s,g}^{\max}\right],g\in G,x\in \left[-0.2,0.2\right],y\in \left[0.05,0.15\right]\right\}$$

As shown in formula 2, where: Ω: set of feasible regions of serve parameters; The upper and lower bounds of each variable are set based on the measured data of competitive table tennis athletes: θ∈[15°,60°]: avoid mistakes caused by too flat/too steep racket surface; h∈[0.15,0.35]: the reasonable height of the ball in accordance with the falling trajectory of the throwing ball; t∈[−0.1,0.1]: cover the effective hitting range before and after the throwing apex; *g* represents the athlete’s technical type, while $v_{s,g}^{\min}$ and $v_{s,g}^{\max}$ represent the lower and upper limits of the swing speed for athletes of that type, respectively. x∈[−0.2,0.2]: limit the touch point to the horizontal effective area of the racket; y∈[0.05,0.15]: limit the contact point to the vertical middle area of the racket.

The lower and upper bounds of swing velocity were determined from the valid serve trials of athletes belonging to the same technical category. Athletes were classified according to their dominant technical characteristics, including speed-oriented, spin-oriented, placement-control, offensive-connection, stability-oriented, and balanced types. For each category, the feasible swing-velocity range was defined according to the observed distribution of valid trials, while isolated extreme values and technically unstable actions were excluded. When the measured upper boundary of a speed-oriented athlete exceeded 15 m/s, the upper constraint was correspondingly extended rather than being truncated at 15 m/s.

After setting the parameter vector and feasible region, the focus of the model turns to screening executable combinations. Among them, the racquet face angle, touch height, hitting time, swing speed and landing point coordinates affect the rotation, arc, rhythm, ball speed and spatial control respectively. By reasonably restricting the range of parameters, the invalid solution can be avoided and the space for individual adjustment of athletes can be reserved.

### 2.5 Service quality evaluation index and multi-objective optimization model construction

The evaluation of serve quality is the key to link technical parameters and optimization algorithm. Speed, rotation, landing point and motion stability jointly determine the service effect, and it is difficult to reflect the competitive value only based on a single index. The research shows that the hitting action is affected by the coordination of the motion chain, and there are differences in the kinematic characteristics of different service types, so the service evaluation should take into account the technical characteristics and tactical needs (Chen et al., 2024; Bańkosz and Winiarski, 2025). In addition, high-quality service does not simply pursue the maximization of ball speed or rotation, but should take into account the balance of speed, rotation, stability, threat and action enforceability. There is a complex synergy between ball speed and rotation (Bańkosz et al., 2025). Therefore, this paper constructs a multi-objective evaluation system including rotation, speed, landing point stability, reception pressure and movement adjustment cost to achieve the unified optimization of maximizing competitive revenue and minimizing movement deviation.

(3) $$F\left(X\right)={\left[f_{1}\left(X\right),f_{2}\left(X\right),f_{3}\left(X\right),f_{4}\left(X\right),f_{5}\left(X\right)\right]}^{T}$$

As shown in Formula 3, where: $f_{1}\left(X\right)=v_{b}\left(X\right)=k_{1}v_{s}\cos\theta +k_{2}h$: ball speed target, $k_{1}/k_{2}$ is the biomechanical fitting coefficient; $f_{2}\left(X\right)=\omega \left(X\right)=k_{3}v_{s}\text{sin}\theta /r$: rotating angular velocity target, *k*_3_ which is the effective spin-transfer coefficient, and *r* is the radius of the table tennis ball; $f_{3}\left(X\right)=1/\text{Var}\left(x,y\right)$: impact point stability target, Var(x,y) which is the variance of impact point coordinates; $f_{4}\left(X\right)=P\left(X\right)=0.8\text{ErrRate}\left(X\right)+0.2\text{PassiveRate}\left(X\right)$: the oppressive target of receiving service, ErrRate is the opponent’s error rate of receiving service, and PassiveRate the opponent’s passive return rate; $f_{5}\left(X\right)=-D\left(X\right)=-\left({∥X-X_{0}∥}_{2}/\max{∥X-X_{0}∥}_{2}\right)$: the movement difficulty target, *X*_0_ is the athlete habit parameter vector and ${∥\cdot ∥}_{2}$ is Euclidean distance.

The coefficients k_1_, k_2_, and k_3_ were not arbitrarily assigned constants. They were treated as empirical fitting coefficients used to connect the measured serve parameters with the corresponding ball-output characteristics. Specifically, k_1_ represents the contribution of the effective component of racket swing velocity to post-impact ball speed, whereas k_2_ represents the correction effect of contact height on ball speed and flight trajectory. These two coefficients were determined from the preliminary calibration of the synchronized measurements of racket swing velocity, racket face angle, contact height, and post-impact ball velocity. The coefficient k_3_ was defined as an effective spin-transfer coefficient estimated from the relationship between the tangential component of racket velocity and the measured angular velocity of the ball. Therefore, k_3_ reflects the combined effects of racket–ball friction, rubber deformation, impact efficiency, and individual stroke characteristics, rather than representing a fixed material friction coefficient. The coefficient ranges were initially restricted according to the measured data and relevant table tennis kinematic studies, and the final coefficient values used in the optimization model were obtained from the experimental calibration dataset.

ErrRate and PassiveRate were calculated from the observed outcomes of the serve–return trials. ErrRate was defined as the proportion of valid serves that directly caused the receiver to make an illegal return or fail to contact the ball successfully. PassiveRate was defined as the proportion of successfully returned serves that resulted in a passive, defensive, or non-attacking return, thereby providing the server with an opportunity to initiate the third-stroke attack. The ErrRate and PassiveRate weights were set at 0.8 and 0.2, respectively, to reflect their different tactical consequences. A direct return error immediately yields a point, whereas a passive return only creates an opportunity for subsequent attack. The weighting therefore reflects tactical outcome severity, while the normalization maximum of 0.45 was determined from the calibration data. Receiver-side state variables, trial allocation, outcome coding, rater agreement, and validation use are detailed in **Supplementary Appendix Table A4**.

#### 2.5.1 Athlete-specific baseline acquisition and score calibration

Before optimization, each athlete completed 20 valid habitual baseline serves without an externally imposed tactical target. Category-specific baselines were calculated for short-backspin, long-backspin, sidespin, and placement-directed serves. Only legal, synchronized, and technically stable trials were retained; 5% of baseline trials were excluded using the 1.5 × IQR criterion followed by video verification. The athlete-specific baseline vector X_0_ was defined as the 20% trimmed mean of racket-face angle, contact height, contact timing, swing velocity, and landing coordinates. Left-handed x-coordinates were mirrored to a right-handed reference.

Within-athlete repeatability was assessed before X_0_ was used in the motor adjustment cost. The ICC values were 0.92 for racket-face angle, 0.89 for contact height, 0.85 for contact timing, 0.94 for swing velocity, 0.88 for the x-coordinate, and 0.90 for the y-coordinate. The coefficients of variation were 3.2% for racket-face angle and 4.5% for swing velocity.

Objective measurements were converted to the reported scales using calibration anchors fixed before validation. Spin intensity was measured in rpm and mapped from 1,000 to 8,000 rpm, while ball speed was mapped from 5 to 25 m/s; values outside these ranges were clipped to 0 or 10. Landing-point stability used radial RMSE from 10 trials per target, with 0 cm as the best anchor and 30 cm as the worst anchor; the score decreased by 1 point for each 3-cm increase in RMSE. Return suppression combined ErrRate and PassiveRate with weights of 0.8 and 0.2 and was normalized against a calibration maximum of 0.45.

Motor adjustment cost was calculated after Z-score standardization of the decision variables with equal weights. The resulting standardized multivariate deviation from X_0_ was rescaled to 0–5, where 0 indicated no adjustment and 5 indicated an extreme adjustment. The comprehensive adaptation index used weights of 0.2 for simulated serve success, 0.4 for return suppression, 0.2 for third-stroke connection, and 0.2 for active scoring. All anchors and thresholds were derived from the 42-athlete calibration set (1,512 trials) and applied unchanged to the 18-athlete validation set (648 trials). Complete calibration rules are provided in **Supplementary Appendix Table A5**.

(4) $$\min F^{\prime }\left(X\right)={\left[-f_{1}\left(X\right),-f_{2}\left(X\right),-f_{3}\left(X\right),-f_{4}\left(X\right),-f_{5}\left(X\right)\right]}^{T}\;s\text{.t}.X\in \Omega$$

As shown in formula 4, where: *F*′(*X*): is transformed into the minimization objective function vector, and the maximization objective is taken as negative; s.t.X∈Ω: constraints to ensure that the parameters are within the feasible range.

### 2.6 Improved particle swarm optimization algorithm design and constraint processing mechanism

After completing the construction of service parameter system and multi-objective evaluation model, the key is to efficiently search the feasible solution in the complex parameter space. The parameters of table tennis serve have the characteristics of continuity, constraint and multi-objective coupling. The parameters affect each other. Different serve types have differences in rotation performance and technical control. Therefore, the optimization algorithm needs to have the ability of multi parameter balanced search (Delumeau et al., 2025). Therefore, this paper introduces dynamic inertia weight and constraint correction mechanism based on standard particle swarm optimization algorithm to improve the adaptability of the algorithm to serve parameter optimization. The research shows that the motion parameters can be quantified and tracked, and the technical optimization should take into account the motion effect, stability and competition applicability (Lyu et al., 2025; Tamaki and Yoshida, 2025). Therefore, the improved PSO algorithm improves the quality of Pareto candidate solutions by dynamically adjusting the global search and local convergence capabilities.

(5) $$v_{i,j}\left(t+1\right)=w\left(t\right)v_{i,j}\left(t\right)+c_{1}r_{1}\left(pbest_{i,j}-x_{i,j}\left(t\right)\right)+c_{2}r_{2}\left(gbest_{j}-x_{i,j}\left(t\right)\right)$$

As shown in formula 5, where: $v_{i,j}\left(t+1\right)$: the velocity update value of the *i* particle at the *j* dimension; $w\left(t\right)=w_{\max}-\left(w_{\max}-w_{win}\right)t/T_{\max}$: dynamic inertia weight, $w_{\max}=0.9$, $w_{\min}=0.4$, *t* is the current iteration number and *T*_max_ the maximum iteration number; $c_{1}=c_{2}=1.5$: learning factor; $r_{1},r_{2}\in \left[0,1\right]$: random number; *pbest_i_*,*_j_*: the individual optimal position of the *i* particle at the *j* dimension; *gbest_j_*: the *j* dimension of the global optimal position; *x_i_*,*_j_*(*t*): the current position of the *i* particle at the *j* dimension.

(6) $$x_{i,j}\left(t+1\right)=\{\begin{array}{ll} lb_{j}+\alpha \left(ub_{j}-lb_{j}\right)\text{rand()} & x_{i,j}\left(t+1\right)<lb_{j}\\ ub_{j}-\alpha \left(ub_{j}-lb_{j}\right)\text{rand()} & x_{i,j}\left(t+1\right)>ub_{j}\\ x_{i,j}\left(t+1\right) & \text{otherwise} \end{array}$$

As shown in formula 6, where $x_{i,j}\left(t+1\right)$: the position of the *i* particle at the *j* dimension is updated; *lb_j_*: lower bound of *j* dimension parameter; *ub_j_*: upper bound of *j* dimension parameter; *α* = 0.1: correction coefficient, which pulls the super boundary particles back to the feasible region; rand(): the random number of the interval (0,1).

To ensure the fairness and reproducibility of the cross-algorithm comparison, all optimization algorithms were implemented in Python 3.9 and executed on the same computer equipped with an Intel Core i9-12900K CPU, 64 GB of memory, and no dedicated GPU acceleration, under the Windows 10 Pro environment. Standard PSO, multi-objective PSO, Genetic Algorithm, NSGA-II, unconstrained modified PSO-Pareto, and the proposed improved PSO-Pareto algorithm were evaluated using the same serve dataset, objective functions, decision-variable boundaries, constraint conditions, and initial sampling space. No parallel acceleration was applied during the runtime comparison.

To ensure a fair comparison, all algorithms used the same population size of 100, a maximum of 200 iterations, and an identical fitness-evaluation budget of 20,000. The same initialization procedure and random seeds were used, and each algorithm was independently repeated 30 times. The termination rule was identical for all algorithms: a run stopped when the maximum iteration number was reached or when the improvement in the best objective value remained below 1 × 10^-4^ for 15 consecutive iterations. Wall-clock time was recorded from initialization to the actual termination point of each run. For the PSO-based algorithms, the acceleration coefficients were set to c_1_ = 1.5 and c_2_ = 2.0; the standard PSO used an inertia weight of 0.7, whereas the improved PSO-Pareto algorithm adopted a dynamic inertia weight decreasing from 0.9 to 0.4. For the Genetic Algorithm and NSGA-II, the crossover probability and mutation probability were set to 0.8 and 0.1, respectively. These unified settings ensured that the differences in convergence and solution quality were primarily attributable to the optimization mechanisms rather than unequal computational resources or search budgets.

### 2.7 Pareto optimal solution set generation and serve parameter combination screening

After completing the search and constraint processing of the improved particle swarm optimization algorithm, the optimization focus turns to the selection of parameter combinations with training value from candidate solutions. Because the service quality is affected by the speed, rotation, stability of the landing point, compression effect and action executability, a single optimal solution is difficult to meet the diverse training needs. Research shows that the selection of service parameters should serve the continuous technical and tactical benefits, rather than only evaluate the single effect (Chen et al., 2025). Therefore, this paper uses Pareto optimal idea to construct the non-dominated solution set, showing the trade-off relationship between different objectives. The comprehensive evaluation study further pointed out that technical optimization needs to be adjusted in combination with multiple indicators, athlete types and competition situations (Chu et al., 2025; Liu et al., 2025; Yin et al., 2025). Pareto solution set can reflect the constraint relationship between parameters, and provide a variety of executable schemes for different training objectives to improve the flexibility and practical value of optimization results.

Based on the Pareto optimality theory, the non-dominated solution and crowding distance are defined *X_a_* dominate *X_b_* if and only if ∀*k*, $F_{k}^{\prime }\left(X_{a}\right)\leq F_{k}^{\prime }\left(X_{b}\right)$ and ∃*k*, $F_{k}^{\prime }\left(X_{a}\right)<F_{k}^{\prime }\left(X_{b}\right)$. Where: *X_a_*, *X_b_* two candidate parameter vectors; $F_{k}^{\prime }\left(X\right)$: the *k* dimension of the objective function vector $F^{\prime }\left(X\right)$; Dominance relationship means *X_a_* that it is not inferior to *X_b_* all goals, and at least one goal is better.

(7) $$CD_{i}=\sum_{k=1}^{5}\frac{F_{k}^{\prime }\left(X_{i+1}\right)-F_{k}^{\prime }\left(X_{i-1}\right)}{F_{k}^{\prime \max}-F_{k}^{\prime \min}}$$

As shown in formula 7, where: *CD_i_*: the crowding distance of the *i* particle; $F_{k}^{\prime }\left(X_{i+1}\right)$, $F_{k}^{\prime }\left(X_{i-1}\right)$: the *k* dimension target value of the i+1/i−1 particle after sorting; $F_{k}^{\prime \max}$, $F_{k}^{\prime \min}$: the global maximum/minimum value of the dimension target value.

**Figure 1:**
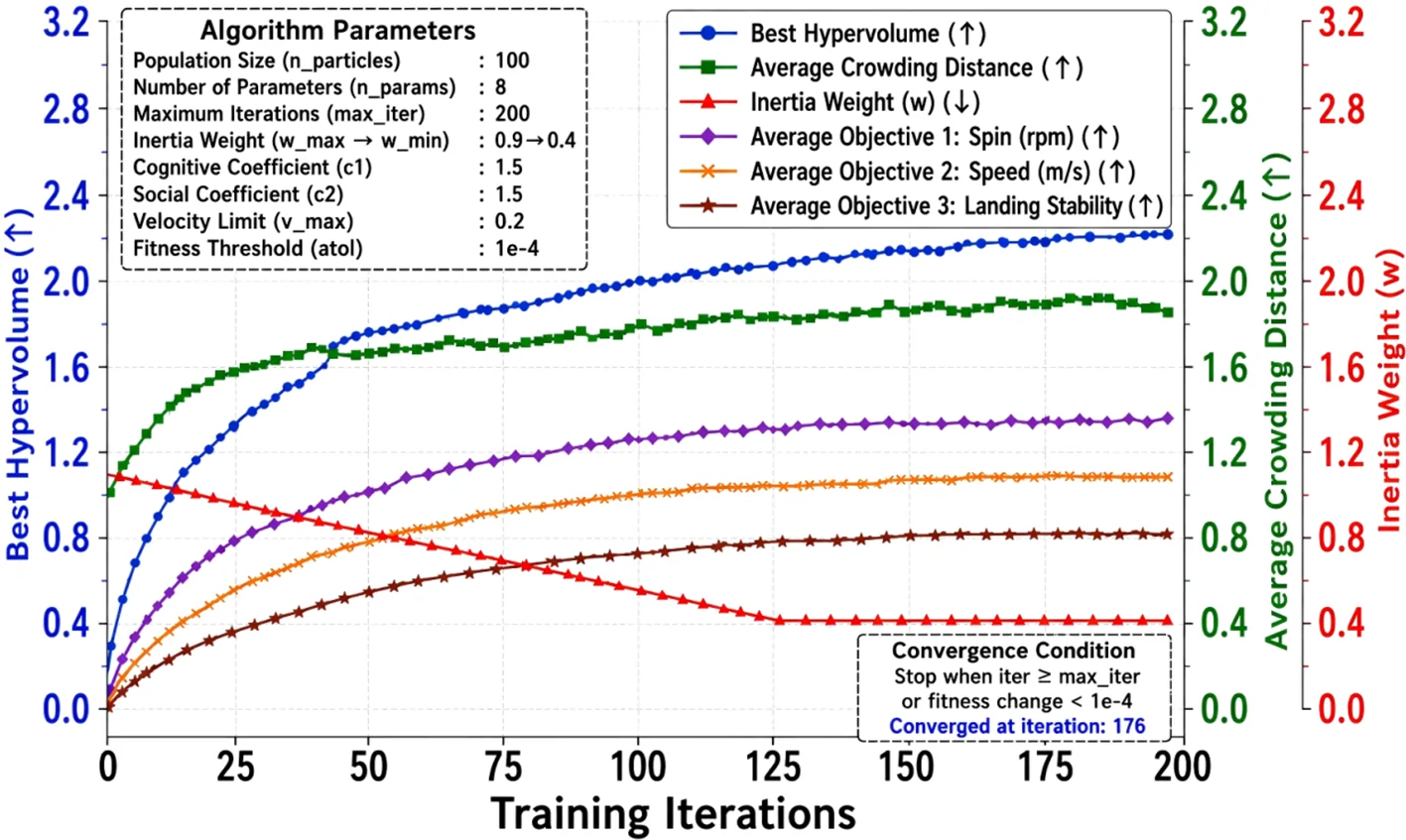
Training curve and iteration curve.

The core algorithm of this study is shown in **Table 1**. **Figure 1** shows the optimization process of the improved PSO Pareto algorithm. The training curve shows that the comprehensive fitness decreases rapidly with iteration and gradually converges. The iterative curve shows that the number of non-dominated solutions increases continuously and then tends to be stable, indicating that the algorithm has good search efficiency, convergence and Pareto solution set retention ability.

**Table 1:** Core algorithm steps.

Step	Explain	Core code
1	Initialize the serve parameter vector (racquet angle, touch height, hit time, swing speed, landing coordinates) and particle swarm position/speed, and the parameter range conforms to the kinematic constraints	pos = np.random.uniform(low=param_bounds[:0], high=param_bounds[:1], size=(n_particles, n_params)) v = np.random.uniform(low=-v_max, high=v_max, size=(n_particles, n_params))
2	Calculate the multi-objective fitness of each particle (rotation score, speed score, landing point stability score, compression effect score, motion adjustment cost)	fitness = compute_multi_objective_fitness(pos)
3	The particles are non dominated sorted to determine the Pareto level, and the crowding distance is calculated to maintain the diversity of solution sets	fronts = non_dominated_sort(fitness) crowding_dist = compute_crowding(fronts, fitness)
4	The dynamic inertia weight is used to update the particle velocity, and combined with the historical optimal/global optimal adjustment position, the out of bounds parameters are constrained and modified	w = w_max - (w_max - w_min)*iter/max_iterv = w*v + c1*np.random.rand()*(pbest-pos) + c2*np.random.rand()*(gbest-pos)
5	Determine whether the maximum number of iterations or convergence conditions are reached. If not, return to step 2	pos = pos + vpos = np.clip(pos, param_bounds[:0], param_bounds[:1])
6	Collect all non dominated particles to generate Pareto optimal solution set	if iter >= max_iter or np.allclose(fitness, prev_fitness, atol=1e-4): break
7	Select the combination of adaptation parameters from the solution set according to the type of athlete (speed/rotation/landing advantage, etc.)	pareto_set = pos[fronts (0)]

To improve the practical interpretability of the Pareto solutions, all retained non-dominated solutions were further classified into six tactical categories according to their dominant objective characteristics: spin-priority, speed-priority, placement-stability, pressure-offense, motion-robustness, and balanced-overall strategies. For each tactical category, the numerical ranges of racket face angle, ball contact height, contact timing, swing velocity, and landing coordinates were extracted directly from the corresponding Pareto solution subset. The minimum and maximum values within each category were used to define the feasible parameter range, Landing coordinates were expressed jointly by the lateral coordinate x and longitudinal coordinate y. All reported ranges satisfied the predefined biomechanical constraints and were retained only when the corresponding solutions met the requirements of technical stability, tactical effectiveness, and motor executability.

The competition-context adaptability analysis was based on scenario simulation rather than real-match statistics. For each competition context, the corresponding Pareto-optimal parameter combinations were selected according to the predefined tactical priorities. Random disturbances were introduced within the observed execution ranges of the athletes, and a simulated serve was considered successful when the generated parameters remained within the feasible region and the predicted landing point reached the target area. The simulated serve success probability was calculated as the proportion of successful trials among 1000 repeated simulations. Receiver-side outcomes used to calibrate and validate the return-suppression objective were obtained from the separate serve–return experiment summarized in **Supplementary Appendix Table A4**. **Table 2** remains a scenario simulation and should not be interpreted as official-match evidence.

**Table 2:** Model-simulated adaptation effects of Pareto-optimal parameter combinations across predefined competition contexts.

Context	Simulated serve success (%)	Return suppression (0–10)	Third-stroke success (%)	Active score rate (%)	Adaptation index (0–10)
Initial Exploratory Stage	85 ± 3	7.5 ± 0.4	60 ± 4	15 ± 2	8.2 ± 0.2
Pre Stalemate Rush Stage	82 ± 4	9.0 ± 0.3	75 ± 3	25 ± 3	8.8 ± 0.2
The opponent’s Weak Reception Stage	80 ± 3	8.5 ± 0.3	65 ± 4	22 ± 2	8.5 ± 0.2
Opponent Backhand Pressure Stage	88 ± 2	7.8 ± 0.2	68 ± 3	18 ± 2	8.4 ± 0.1
Key Score Stage	92 ± 1	7.2 ± 0.3	62 ± 3	12 ± 1	8.6 ± 0.1
Training Consolidation Phase (Rotation First)	78 ± 4	8.8 ± 0.2	58 ± 5	16 ± 2	8.0 ± 0.2

All indicators in this table are model-simulated estimates rather than direct statistics obtained from official matches.

## 3 Results

### 3.1 Participant and dataset characteristics

A total of 2400 serve trials were initially collected from 60 participants. After the exclusion of invalid or incomplete trials, 2160 trials were retained for the final analysis, corresponding to a retention rate of 90.0%. Descriptive analysis showed that the racket face angle, contact height, contact timing, swing velocity, and landing coordinates covered the principal technical ranges observed across the participating athletes, providing an adequate empirical basis for parameter calibration and multi-objective optimization. The separate serve–return validation block generated 3,980 valid trials from 4,320 planned trials (92.1%). Sixty athletes entered the individualized intervention, and 58 completed the posttest. Two athletes withdrew because of mild shoulder strains, and no serious adverse events were recorded.

### 3.2 Performance verification and comparative analysis of improved PSO algorithm

This study found that different optimization algorithms have obvious differences in the search efficiency of serve parameters and the quality of the solution set. As shown in **Table 3**, the improved particle swarm Pareto method performs better in the number of convergence iterations, the optimal target value, the size of the solution set and the hypervolume index. Compared with standard particle swarm optimization algorithm, multi-objective particle swarm optimization algorithm, genetic algorithm and non-dominated sorting genetic algorithm, the improved method can obtain higher quality candidate solution set in less iterations, which shows that the dynamic search mechanism and constraint processing strategy improve the global optimization ability of the algorithm. The average running time showed only a modest numerical increase, which shows that the method achieves a good balance between optimization accuracy and calculation efficiency, and is suitable for the training optimization scenario of continuous variables and multi-objective trade-offs such as table tennis serve parameters. The improved PSO Pareto is located in the upper left region, which shows that its convergence is faster and the target value is higher. At the same time, Pareto solution set size and hypervolume index also perform better.

**Table 3:** Performance comparison of different optimization algorithms.

Optimization algorithm	Number of convergence iterations (Times)	Optimal target value (comprehensive score)	Pareto solution set size (PCS.)	Hypervolume (HV)	Average running time (s)
Standard PSO	120 ± 15	0.65 ± 0.05	15 ± 3	0.42 ± 0.03	12 ± 2
Multi Objective PSO	100 ± 12	0.72 ± 0.06	20 ± 4	0.50 ± 0.04	15 ± 3
Genetic Algorithm	110 ± 14	0.70 ± 0.05	18 ± 3	0.48 ± 0.03	20 ± 4
NSGA-II	90 ± 10	0.75 ± 0.04	25 ± 5	0.55 ± 0.02	18 ± 3
Unconstrained Modified PSO Pareto	85 ± 11	0.78 ± 0.03	28 ± 4	0.60 ± 0.03	16 ± 2
Improved PSO Pareto	70 ± 8	0.82 ± 0.02	35 ± 6	0.68 ± 0.02	14 ± 2

Under the identical termination rule and fitness-evaluation budget, standard PSO converged after 120 ± 15 iterations, whereas the improved PSO-Pareto method converged after 70 ± 8 iterations, corresponding to a 41.7% reduction. The mean time to convergence was 12 ± 2 s and 14 ± 2 s, respectively. Thus, the improved method required fewer iterations, although this was accompanied by a modest increase in computational time.

The experimental results show that the stability differences of different algorithms after repeated operation are prominent, as shown in **Table 4**. The improved particle swarm Pareto method has the highest average value of the target value, the lowest fluctuation of the target value, and the proportion of feasible solutions and the uniformity of the distribution of sets are also better than other comparative algorithms. This shows that this method can not only obtain better single optimization results, but also maintain stable search performance in multiple runs. In contrast, the target values of standard particle swarm optimization and genetic algorithm fluctuate greatly, and the proportion of feasible solutions is relatively low, indicating that they are easily affected by the initial population and search path under complex constraints. The improved PSO Pareto has the best performance in target value, feasible solution proportion and dispersion, with the smallest bubble and the highest position, indicating that its solution set stability and feasibility are superior to other algorithms.

**Table 4:** Evaluation table of repeated operation stability of different algorithms.

Optimization algorithm	Target value mean	Standard deviation of target value	Proportion of feasible solutions (%)	Uniformity of solution set distribution (dispersion)	Stability judgment
Standard PSO	0.65	0.08	75 ± 5	0.15 ± 0.03	difference
Multi Objective PSO	0.72	0.07	80 ± 4	0.12 ± 0.02	commonly
Genetic Algorithm	0.70	0.09	78 ± 3	0.13 ± 0.02	commonly
NSGA-II	0.75	0.05	85 ± 3	0.10 ± 0.01	preferably
Unconstrained Modified PSO Pareto	0.78	0.04	88 ± 2	0.09 ± 0.01	preferably
Improved PSO Pareto	0.82	0.03	95 ± 2	0.07 ± 0.01	excellent

This study found that different modules of the algorithm have different contributions to the overall optimization effect, as shown in **Table 5**. The comprehensive performance evaluation of the completely improved particle swarm Pareto method is the highest, indicating that dynamic inertia weight, constraint correction, non-dominated sorting and crowding distance maintenance together constitute the key basis for improving the performance of the algorithm. After removing any module, the comprehensive performance of the algorithm decreased to varying degrees, and the performance decreased substantially after removing the constraint correction mechanism, indicating that the optimization of serve parameters should not only focus on the search ability, but also ensure that the parameter combination conforms to the real action range and the game executable. Non-dominated sorting and crowding distance maintenance help to maintain the quality and diversity of solution sets, so that the algorithm can provide a variety of alternative service schemes, rather than limited to a single result.

**Table 5:** Ablation validation table of improved PSO algorithm.

Algorithm configuration	Dynamic inertia weight	Constraint correction mechanism	Nondominated sorting	Crowding distance	Comprehensive performance evaluation (1–5 Points)
Standard PSO	no	no	no	no	3.0 ± 0.2
Remove Dynamic Inertia Weight	no	yes	yes	yes	3.5 ± 0.3
Remove Constraint Correction Mechanism	yes	no	yes	yes	3.2 ± 0.2
Remove Non Dominated Sorting	yes	yes	no	yes	4.0 ± 0.2
De Crowding Distance Maintenance	yes	yes	yes	no	3.8 ± 0.3
Complete Improvement of PSO Pareto	yes	yes	yes	yes	5.0 ± 0.1

### 3.3 Pareto optimal serve parameter combination result analysis

**Figure 2:**
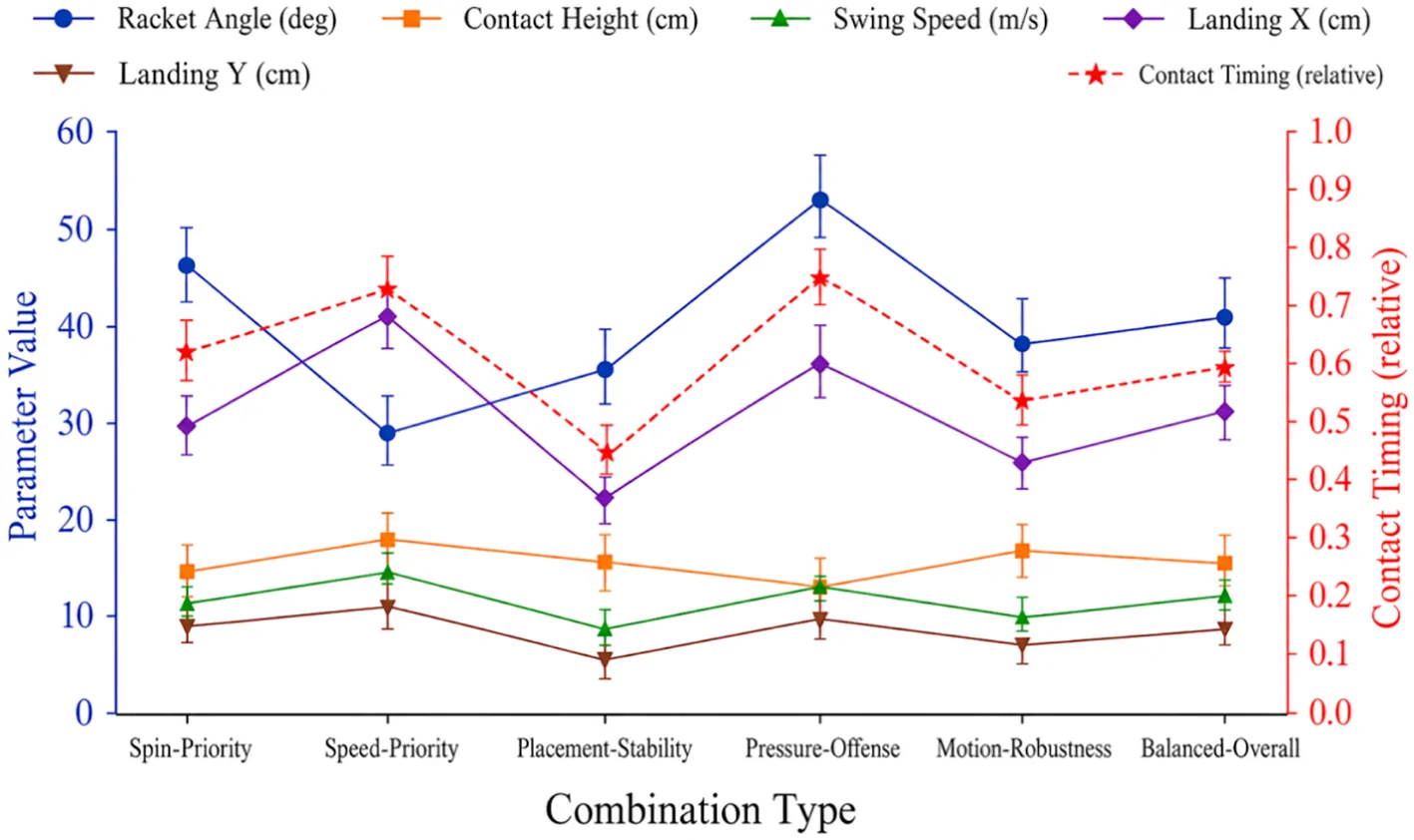
Representative decision-variable profiles of six Pareto-optimal serve strategies.

The experimental results show that the Pareto optimal solution set can form multiple types of service parameter combinations. Different combinations show the characteristics of travel alienation in terms of racket face angle, touch height, hitting time, swing speed and landing coordinates. The rotation priority type emphasizes racquet surface control and rotation manufacturing, while the speed priority type improves ball pressure through higher swing speed; The stable type pays more attention to the height of the ball and the control of the landing point, which is suitable for improving the stability of the service; The pressure attack type has more attack direction in terms of speed, angle and timing, and is suitable for creating active opportunities in the first three boards; The comprehensive equilibrium type maintains relative coordination among multiple parameters. **Figure 2** shows the variation differences of six types of Pareto optimal service parameter combinations in racket face angle, ball touch height, hitting opportunity, swing speed and landing point coordinates, reflecting the parameter trade-off relationship between different service strategies in speed, rotation, stability and attack pressure. The curves illustrate the central parameter tendencies of the six tactical categories, while the complete numerical ranges are reported in **Supplementary Appendix Table A3**.

This study found that the Pareto-optimal serve combinations showed clear functional differentiation across the serve-quality dimensions. As shown in **Table 6**, the spin-priority type had the highest spin score, the speed-priority type had the highest speed score, and the landing-point stability type showed the strongest landing control. The pressure-offense type showed a high return-suppression score but also a relatively high motor adjustment cost, indicating that it may be more suitable for athletes with stronger execution control. The balanced-overall type maintained coordinated performance across spin, speed, landing stability, and return suppression.

**Table 6:** Serve-quality evaluation of Pareto-optimal parameter combinations.

Parameter combination type	Rotation score (0-10)	Speed score (0-10)	Landing-point stability score (0–10)	Return-suppression score (0–10)	Motor adjustment cost (0–5)
Spin-Priority	9.2 ± 0.3	7.5 ± 0.4	8.0 ± 0.2	8.5 ± 0.3	3.5 ± 0.2
Speed-Priority	7.0 ± 0.4	9.5 ± 0.2	7.8 ± 0.3	8.8 ± 0.2	3.2 ± 0.1
Landing-Point Stability	7.8 ± 0.3	7.2 ± 0.3	9.5 ± 0.1	7.5 ± 0.4	2.8 ± 0.1
Pressure-Offense	8.5 ± 0.2	9.0 ± 0.3	8.2 ± 0.2	9.3 ± 0.2	4.0 ± 0.2
Motion-Robustness	8.0 ± 0.3	7.8 ± 0.2	9.0 ± 0.2	8.0 ± 0.3	2.5 ± 0.1
Balanced-Overall	8.5 ± 0.2	8.5 ± 0.2	8.8 ± 0.1	8.7 ± 0.2	3.0 ± 0.1

**Figure 3:**
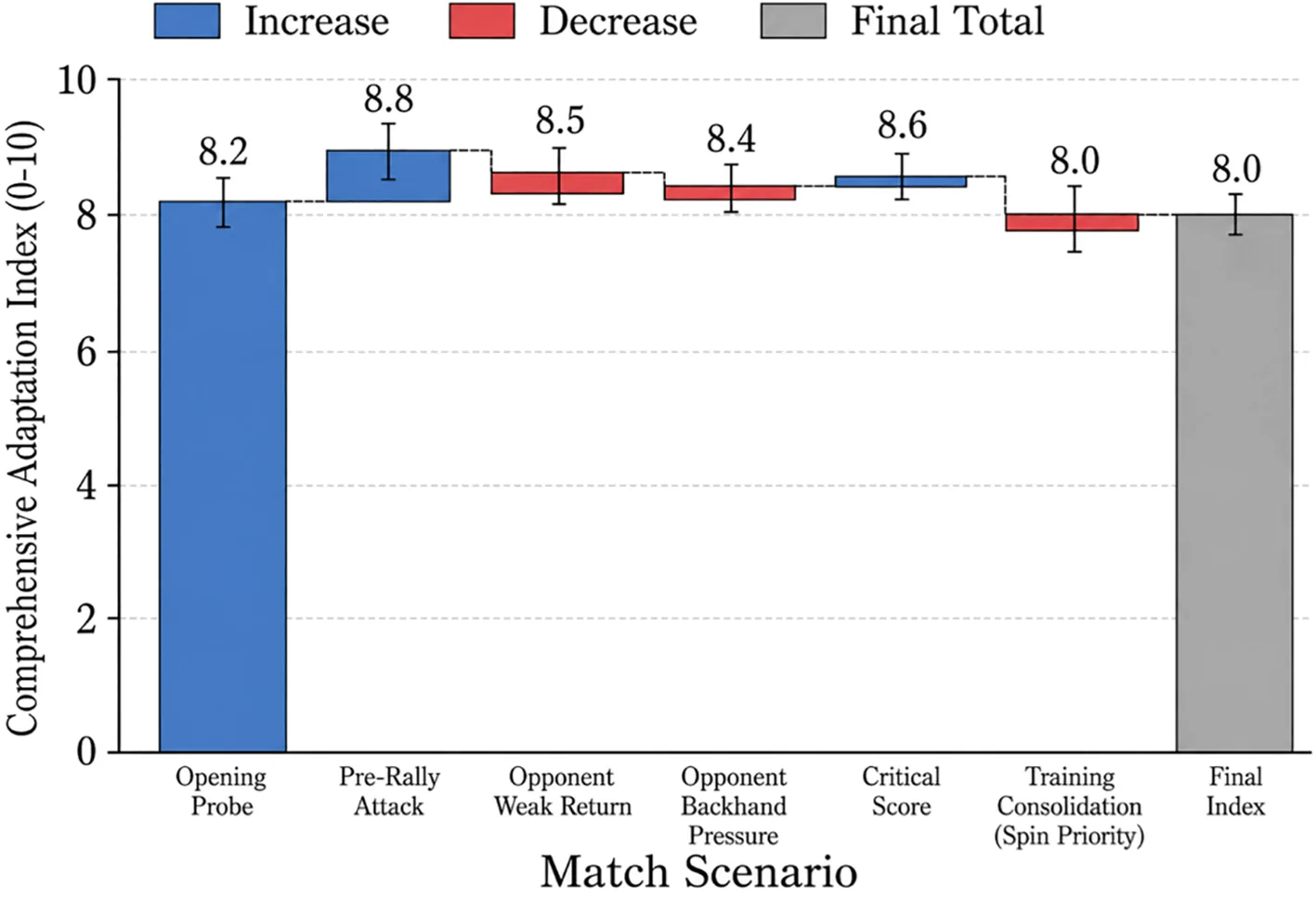
Waterfall analysis chart.

**Table 2** presents model-simulated adaptation estimates for predefined competition contexts. The pre-rally attack context produced the highest simulated return-suppression and third-stroke connection values, whereas the critical-score context favored serve stability. The training-consolidation context emphasized spin control and repeatability. These results illustrate potential context-dependent differences among Pareto-optimal schemes; they do not demonstrate dynamic opponent adaptation or official-match effectiveness (**Figure 3**).

### 3.4 Sensitivity of serve parameters and effect analysis of Individualized Recommendation

**Figure 4:**
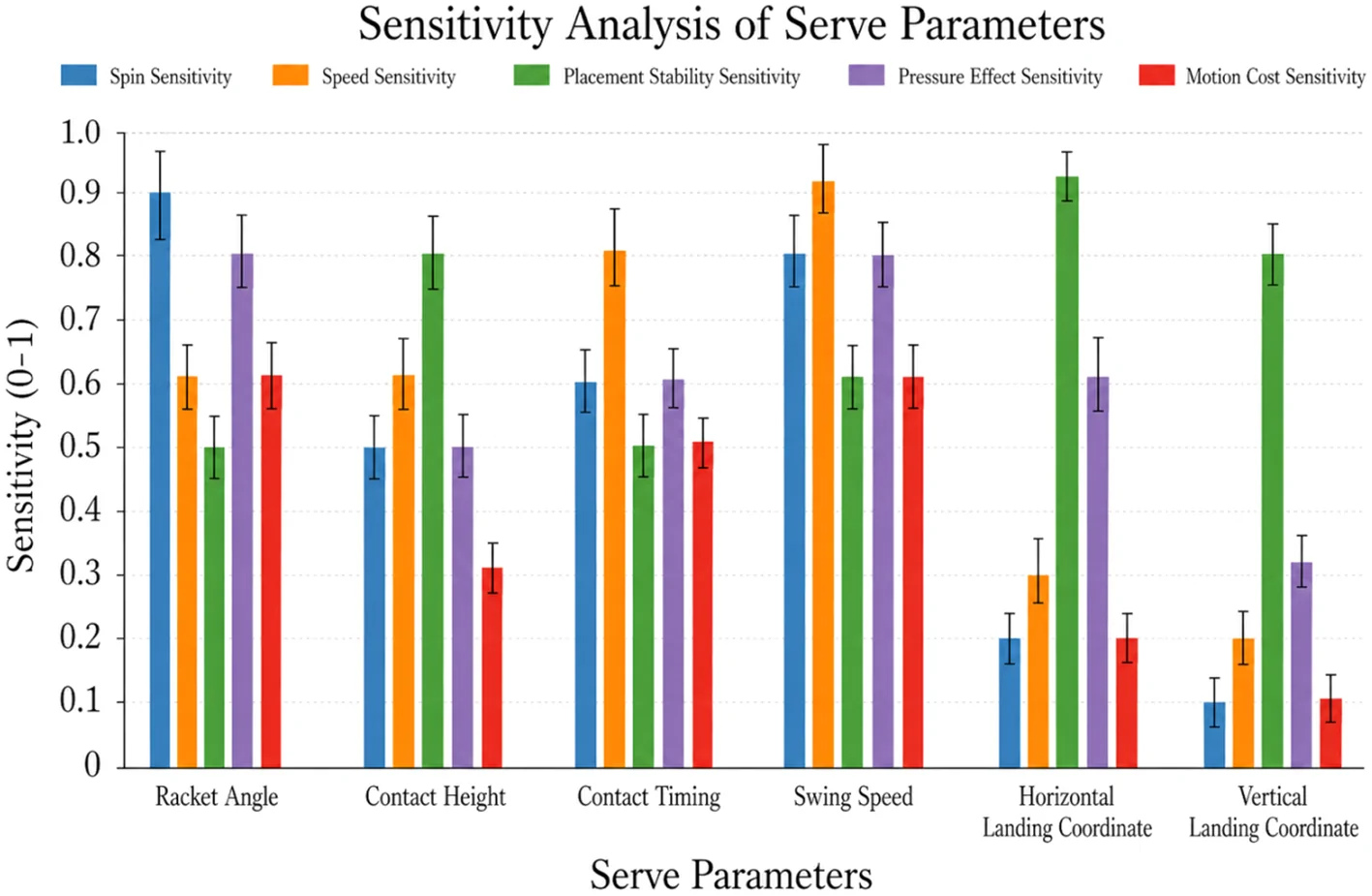
Sensitivity analysis of serve parameters.

The sensitivity analysis showed that serve parameters contributed differently to the five quality indicators. Racket-face angle was most strongly associated with spin and return suppression, while swing velocity was most strongly associated with ball speed and return suppression. The horizontal and longitudinal landing coordinates primarily affected landing-point stability. Contact height and contact timing had moderating effects across speed, stability, and motor adjustment cost. **Figure 4** summarizes these relative sensitivities.

Individualized recommendations showed different descriptive improvement patterns across athlete profiles. As shown in **Table 7**, speed-dominant athletes had the largest numerical increase in serve speed, spin-dominant athletes had the largest increase in spin intensity, and landing-point control athletes achieved the highest landing-point hit rate. Motion-robustness athletes retained the highest movement-stability score, while balanced athletes showed coordinated changes across multiple indicators.

**Table 7:** Individualized serve-recommendation outcomes by athlete type.

Athlete type	Serve-speed gain (%)	Spin-intensity gain (%)	Landing-point hit rate (%)	Movement stability (0–10)	Adaptation index (0–10)
Speed-Dominant	15 ± 2	8 ± 1	88 ± 2	7.5 ± 0.3	9.0 ± 0.2
Spin-Dominant	10 ± 1	18 ± 2	85 ± 3	7.8 ± 0.2	9.2 ± 0.1
Landing-Point Control	12 ± 1	11 ± 1	92 ± 1	8.2 ± 0.2	8.8 ± 0.2
Attack-Connection	14 ± 2	13 ± 1	86 ± 2	7.0 ± 0.3	8.5 ± 0.2
Motion-Robustness	9 ± 1	7 ± 1	90 ± 2	9.0 ± 0.1	8.6 ± 0.1
Balanced-Overall	12 ± 1	12 ± 1	87 ± 2	8.0 ± 0.2	8.7 ± 0.2

The descriptive results showed that the performance indicators of all athlete types increased after the individualized recommendations, as presented in **Table 8**. Improvements were observed in serve quality score, landing-point hit rate, return suppression effect, and third-stroke connection rate. Speed-dominant and attack-connection athletes showed relatively larger numerical increases in attack-related indicators, whereas spin-dominant and landing-point control athletes showed more pronounced improvements in spin quality and placement accuracy. Motion-robustness athletes exhibited relatively smaller changes in attacking indicators but maintained improvements in overall serve quality and execution stability. Because the current analysis focused on descriptive before-and-after comparisons and did not include inferential statistical testing, these changes are interpreted as observed improvement trends rather than statistically significant effects. The recommendation delivery, training dose, adherence, and pre–post assessment protocol underlying **Tables 7**, **8** are summarized in **Supplementary Appendix Table A6**. The pre–post summaries were calculated from the 58 athletes who completed the intervention using available-case descriptive analysis.

**Table 8:** Descriptive comparison of serve performance before and after individualized recommendations.

Athlete type	Serve quality (0–10)	Landing-point hit rate (%)	Return suppression (%)	Third-stroke success (%)	Δ
Speed-Dominant Athlete	7.5 ± 0.4 → 8.8 ± 0.2	82 ± 3→88 ± 2	78 ± 4 → 85 ± 3	65 ± 5 → 75 ± 4	Yes
Spin-Dominant Athlete	8.0 ± 0.3 → 9.2 ± 0.2	78 ± 4 → 85 ± 2	80 ± 3→88 ± 2	68 ± 4 → 72 ± 3	Yes
Landing-Point Control Athlete	7.2 ± 0.4 → 8.5 ± 0.3	85 ± 3 → 92 ± 2	75 ± 4 → 80 ± 3	62 ± 5→68 ± 4	Yes
Attack-Connection Athlete	7.8 ± 0.3 → 8.7 ± 0.2	80 ± 4 → 86 ± 2	82 ± 3 → 88 ± 2	70 ± 4 → 78 ± 3	Yes
Motion-Robustness Athlete	7.0 ± 0.4 → 8.2 ± 0.3	88 ± 2 → 93 ± 1	70 ± 5 → 75 ± 4	60 ± 5 → 65 ± 4	Yes
Balanced-Overall Athlete	7.6 ± 0.3 → 8.6 ± 0.2	83 ± 3 → 89 ± 2	77 ± 4 → 83 ± 3	66 ± 5 → 73 ± 4	Yes

The values represent descriptive before-and-after changes. No inferential statistical significance test was conducted; therefore, the reported changes should not be interpreted as statistically significant effects.

## 4 Discussion

The improved PSO-Pareto method enhanced algorithm performance through dynamic inertia weighting and constraint correction. Compared with standard PSO, the improved PSO-Pareto method reduced the convergence iteration count by 41.7% under the same termination conditions and evaluation budget, while the mean convergence time increased modestly from 12 ± 2 s to 14 ± 2 s. The feasible-solution proportion increased from 75% ± 5% to 95% ± 2%. In the ablation analysis, removing the constraint-correction mechanism reduced the comprehensive performance score from 5.0 to 3.2, supporting its role in maintaining parameter feasibility. These comparisons indicate numerically better convergence, feasibility, and solution coverage under the unified computational settings (**Table 9**).

**Table 9:** Summary of performance improvements introduced by the proposed PSO-Pareto mechanisms.

Optimization dimension	Traditional algorithm problem	Improvement measures	Improvement effect (compared with standard PSO)
Convergence efficiency	Premature convergence and many iterations	Dynamic inertia weight	41.7% reduction in convergence times
Feasible solution ratio	Too many out-of-bound solutions and low practicability	Constraint correction mechanism	Increase the proportion of feasible solutions by 20%
Diversity of solution sets	Single solution, insufficient coverage	Non-dominated sorting + crowding distance	Pareto-set size increased by 133%
stability	The results fluctuated greatly and the repeatability was poor	Multi mechanism collaborative optimization	Standard deviation of target value reduced by 62.5%

The Pareto solution set provided multiple tactically distinct alternatives rather than a single optimum. Non-dominated sorting and crowding-distance maintenance produced a solution set of 35 ± 6 candidates spanning six tactical categories. The interval-based recommendations in **Supplementary Appendix Table A3** are intended to accommodate natural execution variability rather than prescribe single-degree angles or centimeter-level targets. The empirical conclusions of this study are based on measured serve kinematics, 3,980 valid serve–return trials, and the descriptive pre–post outcomes of the 58 athletes who completed the intervention. In contrast, the context-specific values in **Table 2** were generated by scenario simulation and should not be interpreted as actual match outcomes. These estimates depend on predefined tactical priorities, disturbance ranges, and success criteria; therefore, they reflect performance only under the tested assumptions and require further validation using sequential match data. In addition, the sample size, competitive-level distribution, and duration of follow-up should be expanded in future studies. Larger longitudinal datasets integrating high-speed vision, wearable sensors, and intelligent table-recording systems would support external validation and dynamic receiver-aware modeling.

The individualized recommendation results were evaluated primarily through descriptive before-and-after comparisons. Although consistent numerical improvements were observed across the athlete categories, inferential statistical tests were not performed in the present analysis. Therefore, the findings should be interpreted as preliminary evidence of improvement trends, and future studies should use larger paired samples and formal statistical testing to verify the magnitude and significance of the recommendation effects.

## 5 Conclusions

This study constructs a multi-objective optimization framework for table tennis serve parameters by integrating a multidimensional quality-evaluation system with an improved particle swarm optimization algorithm. With racket-face angle, ball contact height, contact timing, swing velocity, and landing coordinates as decision variables, serve quality is evaluated across spin intensity, ball speed, landing stability, return-suppression effect, and motor adjustment cost. The improved PSO-Pareto method showed favorable numerical performance in convergence efficiency, solution diversity, distribution uniformity, and feasible-solution proportion. The framework generated six interval-based tactical strategies and showed descriptive pre–post improvements across athlete profiles. These findings support the potential value of the method for refined and individualized serve training, while real-match dynamic adaptation requires further validation.

## Data Availability

The original contributions presented in the study are included in the article/**Supplementary Material**. Further inquiries can be directed to the corresponding author.
