# Supplementary material for: Multi-objective optimization of table tennis serve kinematic parameters using an improved particle swarm optimization algorithm
Source: Front Physiol. 2026 Sep 4;17:1932912. doi: 10.3389/fphys.2026.1932912 (PMC13585532; doi:10.3389/fphys.2026.1932912)
Supplement: Supplementary file 1 [file Table1.docx]

# Appendix

Table A1. Demographic and training characteristics of the main server cohort

| Characteristic | Value |
| --- | --- |
| Number of participants, n | 60 |
| Male/female, n | 36 / 24 |
| Age, years | 21.4 ± 2.8 |
| Height, cm | 172.5 ± 8.3 |
| Body mass, kg | 65.2 ± 9.6 |
| Training experience, years | 8.6 ± 3.2 |
| Competitive classification, n |  |
| National-level | 8 |
| First-class | 22 |
| Second-class | 30 |
| Dominant hand, n |  |
| Right-handed | 52 |
| Left-handed | 8 |
| Valid serve trials, n | 2160 |

Table A2. Experimental equipment and data-acquisition settings

| Measurement item | Equipment model and manufacturer | Acquisition setting | Measurement accuracy or calibration |
| --- | --- | --- | --- |
| Racket trajectory | Phantom VEO 710, Vision Research, USA | 500 fps; 1280 × 800 pixels; shutter 1/2000 s | Spatial accuracy: 1.0 mm (post-calibration) |
| Ball trajectory | Phantom VEO 710, Vision Research, USA | 500 fps; 1280 × 800 pixels; shutter 1/2000 s | Spatial accuracy: 2.0 mm (post-calibration) |
| Swing velocity | Xsens MTw Awinda, Xsens Technologies, Netherlands | 100 Hz; 3D accelerometer ±160 m/s², gyroscope ±2000°/s | Angular velocity accuracy: ±0.5°/s |
| Ball velocity | Stalker Sport 2, Applied Concepts, USA | 34.7 GHz Doppler radar; 100 Hz | ±0.1 m/s |
| Ball spin | High-speed camera-based marker-tracking method (Phantom VEO 710) | 500 fps; retro-reflective markers (diameter 6 mm) on ball surface | Spin rate accuracy: ±5 rpm |
| Landing coordinates | Qualisys Track Manager (QTM), Qualisys, Sweden | 500 Hz; 4-camera marker-based tracking | Spatial accuracy: 0.5 cm |
| Device synchronization | Qualisys Sync Unit, Qualisys, Sweden | Hardware trigger; TTL pulse synchronization | Inter-device temporal offset: < 0.1 ms |
| Spatial calibration | 3D calibration frame (Peak Performance Technologies, USA) | 24-point calibration wand; dynamic calibration prior to each session | Mean calibration error: 0.3 cm |
| Data-processing software | MATLAB R2023b, MathWorks, USA | 4th-order Butterworth low-pass filter; cutoff frequency 12 Hz; Z-score normalization | — |

Table A3. Numerical ranges of decision variables for six Pareto-optimal serve strategies

| Tactical strategy | Racket face angle (°) | Ball contact height (m) | Contact timing (relative value) | Swing velocity (m/s) | Landing coordinates (m), x and y |
| --- | --- | --- | --- | --- | --- |
| Spin-priority | [42.0–48.0] | [0.35–0.50] | [0.02–0.08] | [10.0–13.0] | x: [−0.10–0.10]; y: [0.30–0.80] |
| Speed-priority | [30.0–38.0] | [0.40–0.55] | [0.00–0.05] | [13.0–17.0] | x: [−0.08–0.12]; y: [0.70–1.50] |
| Placement-stability | [38.0–45.0] | [0.45–0.60] | [−0.05–0.02] | [8.0–11.0] | x: [−0.05–0.05]; y: [0.20–0.60] |
| Pressure-offense | [35.0–42.0] | [0.30–0.45] | [−0.08–0.00] | [14.0–18.0] | x: [−0.15–0.15]; y: [0.50–1.80] |
| Motion-robustness | [40.0–48.0] | [0.40–0.55] | [−0.03–0.03] | [9.0–12.0] | x: [−0.05–0.05]; y: [0.30–0.70] |
| Balanced-overall | [38.0–46.0] | [0.38–0.52] | [−0.02–0.05] | [11.0–14.0] | x: [−0.08–0.08]; y: [0.40–1.00] |

Table A4. Serve–return validation protocol and receiver-side outcome coding

| Item | Standardized procedure | Recorded variable or operational definition | Study-specific value |
| --- | --- | --- | --- |
| Participants and roles | Servers were drawn from the main athlete sample. Receivers completed a separate serve–return validation block. | Server and receiver demographic and competitive characteristics; role separation. | Receivers: n = 18 (10 males and 8 females); age, 21.8 ± 2.5 years; 4 national-level, 8 first-class, and 6 second-class athletes. No athlete served in both roles. |
| Server–receiver pairing | Pairings were standardized and randomized to reduce systematic opponent effects. | Pairing matrix, number of receivers faced by each server, and order of pairings. | Each server faced 3 randomly assigned receivers; 180 pairings in total. |
| Serve conditions and trial volume | The serve–return block used the same serve categories as the main protocol unless otherwise specified. | Trials per serve category, trials per server–receiver pair, total collected trials, and retained valid trials. | 6 trials per serve category per pairing; 4,320 planned trials; 3,980 valid trials retained (92.1%). |
| Randomization and information control | Serve order and target areas were randomized. Receiver knowledge of serve type and target was controlled. | Randomization method; whether receivers were blinded to spin/placement; rest intervals. | Blocked randomization; receivers blinded to spin using an opaque screen; 30-s inter-trial rest and 3-min inter-block rest. |
| Receiver-side state variables | Receiver posture and movement context were recorded for stratified validation. | Initial ready position, receiving side, distance from table, lateral movement range, and tactical anticipation category. | Three-dimensional ready posture; forehand/backhand side; 0.5–1.0 m from the table; lateral range, 0–1.2 m; anticipation: 0 = no cue, 1 = partial cue, and 2 = full cue. |
| Direct return error | A direct return error was coded when the receiver failed to contact the ball, made an illegal return, or failed to return the ball to the opponent’s court. | ErrRate numerator and denominator; treatment of lets and invalid serves. | ErrRate = direct errors divided by valid serves after excluding lets; denominator, 3,980 serves. |
| Passive return | A passive return was coded when the serve was legally returned but the return was defensive or non-attacking and created a third-stroke attacking opportunity for the server. | PassiveRate numerator and denominator; operational criteria distinguishing passive and active returns. | Passive return: attacking intent below 30%, a high-arc return, or a forced lob; denominator, successful returns. |
| Third-stroke connection | The outcome was recorded when the server executed the planned third-stroke attack after the receiver’s return. | Successful connection, failed connection, and no-opportunity categories. | Success: the server attacked within 2 shots after the return; failure: forced error or passive block; no opportunity: the return had already won the rally. |
| Video coding and adjudication | Serve–return outcomes were coded from synchronized recordings by independent qualified assessors. | Number and qualifications of assessors; independent coding; disagreement-resolution procedure. | Two ITTF-certified coaches coded independently; disagreements were resolved by consensus. |
| Reliability and validation use | Coding reliability was assessed before final analysis, and athlete-level separation was maintained between calibration and validation data. | Cohen’s κ/weighted κ or ICC; calibration/validation split; prevention of athlete-level leakage. | Cohen’s κ = 0.88 for ErrRate and 0.82 for PassiveRate; calibration, n = 42 athletes; validation, n = 18 athletes. |

Table A5. Athlete-specific baseline acquisition and score calibration rules

| Component | Procedure | Reporting requirement | Study-specific value |
| --- | --- | --- | --- |
| Habitual-serve baseline block | Athletes performed habitual serves without an externally imposed tactical target before individualized optimization. | State the number of valid baseline trials per athlete and whether a separate baseline was calculated for each serve category. | 20 valid baseline trials per athlete; separate category-specific baselines for short-backspin, long-backspin, sidespin, and placement-directed serves. |
| Baseline screening | Only legal, synchronized, technically stable trials were retained; missing and extreme values were removed using the prespecified quality-control procedure. | State whether 1.5 × IQR, video verification, or another screening rule was applied. | The 1.5 × IQR rule was applied; 5% of trials were excluded after video verification. |
| Definition of X₀ | The athlete-specific baseline vector was obtained from the central tendency of racket-face angle, contact height, contact timing, swing velocity, and landing coordinates. | Specify mean, median, or trimmed mean and whether left-handed coordinates were mirrored. | A 20% trimmed mean was used; left-handed x-coordinates were mirrored to a right-handed reference. |
| Baseline reliability | Within-athlete repeatability was assessed before the baseline was used in the motor adjustment cost. | Report ICC and/or coefficient of variation for each component of X₀. | ICC: angle = 0.92, height = 0.89, timing = 0.85, velocity = 0.94, x = 0.88, and y = 0.90. CV: angle = 3.2% and velocity = 4.5%. |
| Spin-intensity score | Objective ball-spin measurements from the calibration dataset were converted to the 0–10 scale; higher values represented stronger spin. | Report the physical unit, low/high anchors, interpolation rule, and clipping of values outside the calibration range. | Unit, rpm; low anchor, 1,000 rpm; high anchor, 8,000 rpm; linear interpolation; values clipped to 0–10. |
| Ball-speed score | Radar-measured post-impact ball speed was converted to the 0–10 scale; higher values represented greater speed. | Report the low/high calibration anchors and treatment of out-of-range values. | Low anchor, 5 m/s; high anchor, 25 m/s; linear interpolation; values clipped to 0–10. |
| Landing-point stability score | Repeated landing-coordinate dispersion was converted to the 0–10 scale; lower dispersion corresponded to a higher score. | Report the dispersion metric, target area, low/high anchors, and number of trials used. | Radial RMSE; 0–30 cm anchors; 10 trials per target; the score decreased by 1 point per 3-cm increase in RMSE and was clipped to 0–10. |
| Return-suppression score | Observed ErrRate and PassiveRate from the serve–return experiment were combined using the manuscript’s stated weighting and rescaled to 0–10. | Report the exact denominators, weights, calibration anchors, and handling of missing return opportunities. | ErrRate weight = 0.8 and PassiveRate weight = 0.2, reflecting the greater tactical consequence of a direct return error; calibration maximum = 0.45; final score clipped to 0–10. |
| Motor adjustment cost | Deviation from X₀ was evaluated after accounting for differences in variable units and natural within-athlete variability; lower values indicated easier execution. | Report the standardization method, variable weights, low/high anchors, and final 0–5 interpretation. | Z-score standardization with equal weights; standardized multivariate distance rescaled to 0–5; 0 = no adjustment and 5 = extreme adjustment. |
| Comprehensive adaptation index | The index summarized simulated serve success, return suppression, third-stroke connection, and active scoring for the predefined context analysis. | Report component weights, whether weights sum to one, and any sensitivity analysis. | Weights: serve success = 0.2, return suppression = 0.4, third-stroke connection = 0.2, and active scoring = 0.2; sum = 1.0. |
| Calibration and validation separation | All score anchors and thresholds were derived from the calibration dataset and then applied unchanged to the validation dataset. | Report athlete and trial counts in each partition. | Calibration: 42 athletes and 1,512 trials; validation: 18 athletes and 648 trials. |

Table A6. Individualized recommendation intervention and pre–post evaluation protocol

| Element | Implementation | Information required for reproducibility | Study-specific value |
| --- | --- | --- | --- |
| Participants and grouping | Athletes receiving individualized recommendations were grouped by their dominant technical profile. | Number of athletes in each profile and whether the grouping rule was prespecified. | n = 60; speed-dominant = 12, spin-dominant = 12, landing-point control = 10, attack-connection = 10, motion-robustness = 8, and balanced-overall = 8. The grouping rule was prespecified. |
| Pretest | Baseline serve performance was assessed under the same standardized environment and serve categories used for posttest. | Number of trials per outcome, receiver matching, and timing relative to the intervention. | 40 trials per athlete; the same receiver pool as in Table A4; pretest conducted 1 week before the intervention. |
| Recommendation delivery | Each athlete received a Pareto-optimal parameter range selected according to baseline characteristics and tactical priority. | Who selected the final solution, how recommendations were explained, and whether coaches were involved. | Algorithm–coach consensus; visual dashboards; two coaches were consulted. |
| Training duration and frequency | Athletes practiced the individualized serve recommendations during a structured intervention period. | Total intervention duration, sessions per week, and session duration. | 4 weeks; 3 sessions per week; 45 min per session. |
| Practice dose | Each session included a standardized number of recommendation-specific serves with scheduled rest. | Serves per session, block structure, rest intervals, and feedback frequency. | 80 serves per session; 4 blocks of 20 serves; 30-s rest between blocks; real-time kinematic feedback after each block. |
| Adherence and protocol deviations | Attendance and completed serve volume were recorded; deviations were documented. | Adherence threshold, mean attendance, protocol deviations, and exclusions. | Adherence threshold ≥80%; mean attendance = 95%; two athletes withdrew because of injury. |
| Posttest | Post-intervention outcomes were assessed using the same equipment, environment, target areas, and receiver conditions as the pretest. | Timing after the final session and whether assessors were blinded to test occasion. | 3 days after the final session; assessors were blinded to pretest/posttest status. |
| Primary outcomes | Serve quality, landing-point hit rate, return-suppression effect, and third-stroke connection success were evaluated. | Operational definition and denominator for each outcome reported in Tables 7 and 8. | 40 valid trials per athlete; denominator = valid trials per athlete. |
| Receiver-side control | The same receiver pool or a standardized receiver-matching procedure was used at pretest and posttest. | Receiver identity/level matching, posture and receiving-side control, and order randomization. | The same 18 receivers were matched by level; posture was standardized; order was randomized. |
| Statistical handling | The present manuscript reports descriptive before–after changes. Any inferential test should be reported only when supported by athlete-level paired data. | State the final analytic approach, missing-data handling, confidence intervals/effect sizes if calculated, and multiplicity adjustment if applicable. | Descriptive changes only; no inferential tests were performed; missing trial-level data were below 2%. |
| Safety and adverse events | Pain, injury, or discontinuation during the intervention was monitored. | Number and type of adverse events and withdrawals. | Two mild shoulder strains; two withdrawals; no serious adverse events. |
